# From Health Literacy to Self-Care: Contributions of the Specialist Nurse in Rehabilitation Nursing

**DOI:** 10.3390/ijerph19137767

**Published:** 2022-06-24

**Authors:** Marina do Rosário Jesus Dias, Ana da Conceição Alves Faria, Maria Salomé Martins Ferreira, Fabiana Faleiros, André Novo, Maria Narcisa Gonçalves, Carla Gomes da Rocha, Paulo João Figueiredo Cabral Teles, Marlene Patrícia Ribeiro, João Miguel Almeida Ventura da Silva, Olga Maria Pimenta Lopes Ribeiro

**Affiliations:** 1 Surgery Department, Centro Hospitalar de Vila Nova de Gaia/Espinho, 4434-502 Vila Nova de Gaia, Portugal; marina_dias91@hotmail.com; 2 Institute of Biomedical Sciences Abel Salazar, University of Porto, 4050-313 Porto, Portugal; anacafaria85@gmail.com (A.d.C.A.F.); enf.joao.ventura@gmail.com (J.M.A.V.d.S.); 3 North Region Health Administration, 4000-447 Porto, Portugal; 4 Escola Superior de Saúde do Instituto Politécnico de Viana do Castelo, 4900-314 Viana do Castelo, Portugal; salomeferreira@ess.ipvc.pt; 5 Ribeirão Preto College of Nursing, University of São Paulo, Ribeirão Preto 14040-902, Brazil; fabifaleiros@eerp.usp.br; 6 CINTESIS@RISE, 4200-450 Porto, Portugal; andrenovo@gmail.com; 7 Escola Superior de Saúde do Instituto Politécnico de Bragança, 5300-253 Bragança, Portugal; 8 Nursing Department, Nursing School of Porto, 4200-072 Porto, Portugal; mnarcisa@esenf.pt; 9 Institute of Health, School of Health Sciences, HES-SO Valais-Wallis, 1950 Sion, Switzerland; carla.gomesdarocha@hevs.ch; 10 School of Economics, University of Porto, LIAAD-INESC Porto LA, 4200-465 Porto, Portugal; pteles@fep.up.pt; 11 Emergency Department, Centro Hospitalar do Tâmega e Sousa, 4560-136 Penafiel, Portugal; 72601@chts.min-saude.pt; 12 Medicine Department, Centro Hospitalar Universitário de São João, 4200-319 Porto, Portugal

**Keywords:** disease prevention, health literacy, health promotion, nursing, rehabilitation, self-care

## Abstract

(1) Background: Initiatives aimed at assessing and intervening in health literacy have the potential to promote adherence to self-care behaviours, which is the main focus of intervention by rehabilitation nurses. Thus, the objectives were to analyse the level of health literacy of working-age citizens and identify priority areas for intervention by rehabilitation nurses. (2) Methods: Quantitative, correlational and cross-sectional study, conducted in a multinational company, with the participation of 161 workers. The data were collected between 14 April and 7 May 2021, using a self-completion questionnaire composed of sociodemographic and clinical characterization and the European Health Literacy Survey, following a favourable opinion from the Ethics Committee and the company’s management. (3) Results: Overall, low to moderate literacy scores were predominant. Age and education were significantly associated with literacy scores. Workers with higher levels of health literacy had no diagnosed illnesses, took less medication, reported less sadness, fewer memory changes and less muscle and joint pain. (4) Conclusions: The fact that higher levels of health literacy trigger self-care behaviours and, consequently, fewer health problems reinforces the need for rehabilitation nurses to invest in this area.

## 1. Introduction

The low levels of health literacy observed in Portugal led the Portuguese Government to define Health Promotion as a priority in 2016 through the creation of the National Programme for Health, Literacy and Self-Care [1]. Subsequently, in 2018, it created the Action Plan for Health Literacy 2019–2021, with a view to an approach throughout the life cycle, intergenerational, promoting informed choices by citizens [2].

In recent decades, investment in health literacy has played a key role in society, granting citizens a more active role with regard to health and healthcare [3].

Although the concept of health literacy was first used in the 1970s, it was only in the late 1990s that the first definitions of the concept emerged, and since then, it has been evolving. From a definition related to the performance of certain tasks, from a completely individual perspective, it evolved to a concept that incorporates not only the personal but also the social component of the individual, assuming the ability to make informed decisions in their daily lives, taking responsibility for them [3].

In order to bridge some gaps in the understanding of the concept, authors suggested a conceptual model for the design and operationalisation of health literacy [4]. This model highlights the main dimensions of health literacy, namely Health Care, Disease Prevention and Health Promotion, showing which proximal factors (personal and situational determinants) and distal factors (social factors and environmental determinants) have an impact on it and the type of skills needed in the process to *Access*, *Understand*, *Appraise* and *Apply* health information.

Studies highlight that a low level of health literacy is a risk factor for various pathological processes, while adequate levels of health literacy appear to result in improvements in people’s health status. When compared to adequate health literacy, inadequate health literacy is strongly linked to low knowledge or understanding of both health care services and health outcomes themselves and may also be associated with a high likelihood of hospitalisation and low uptake of health promotion and disease prevention services [3,5].

The analysed literature on literacy promotion interventions to improve health outcomes shows that there are a variety of interventions with significant physical, mental and social health outcomes. Specific health literacy promotion programmes and/or materials may be effective strategies [6].

Studies on the assessment of health literacy in different countries and age groups using a common, culturally adapted instrument, the European Health Literacy Survey (HLS-EU), are essential to assess the levels of health literacy in the population [7] and, consequently, to define intervention strategies.

In the study conducted in Portugal, with 1004 participants aged ≥ 16 years, the analysis of the results from the application of the Portuguese version of the HLS-EU revealed low levels of health literacy, which were particularly worrying in the Health Promotion sub-index, where 60.2% of the respondents had a problematic or inadequate level of literacy. Moreover, according to the author’s perspective, taking into account that citizen-centred health care should include the promotion of health literacy, the facilitation of access to health information by the citizen, as well as shared decision-making, there is still a long way to go [7].

In fact, considering that one of the main objectives of the investment in health literacy is to empower citizens as active managers of their health condition and of the community in which they live, with the possibility of interacting with health professionals, the identification of priority areas of intervention is urgent. These assumptions are in line with a set of specific competencies of the Specialist Nurse in Rehabilitation Nursing (SNRN) [8], as well as with the dimensions included in the quality standards recommended by the Portuguese Nurses’ Association, more specifically regarding the descriptive statements “health promotion” and “prevention of complications” [9].

Following a consistent practice with theoretical nursing frameworks [10], SNRNs, focused on the developmental or health/illness transitions experienced by people, are in a privileged position to promote health literacy, ensuring better use of health services, greater ease of communication with health professionals, and better adherence to preventive behaviours and attitudes. Aware of the impact of self-care actions (the main focus of their intervention) on health status, these specialist nurses have been investing in empowering people to manage their health projects, delaying, through health promotion actions, the onset of complications associated with pathological processes and/or the aging process itself [11,12].

In studies that have been carried out in the country, especially with older people [11,13,14], SNRNs have found that an investment in health literacy in earlier age groups would have the potential to prevent some complications later in life. In Portugal, although such investment is being made, namely among the child and adolescent population [15,16], weaknesses are denoted in adulthood.

In fact, although it is consensual that the investment in health literacy should occur as early as possible in childhood/adolescence [17], which has been evident in programmes already implemented in the country [15,16], it is known that, in the current context, the adult population has not had access to this type of interventions. This fact justifies the investment in actions to promote health literacy in this age group.

Therefore, the objectives of this study were to analyse the level of health literacy among working-age citizens and to identify priority areas for intervention by rehabilitation nurses.

## 2. Materials and Methods

This is a correlational and cross-sectional study conducted in a multinational company based in Northern Portugal. The study population refers to all workers who performed their activity in the company in question, which corresponds to a total of 320 workers. Using a non-probability convenience sampling technique, we obtained the participation of 161 workers, i.e., 50.3% of the population. According to the defined criteria, workers who were on leave for any reason and those who were working outside the head office were excluded.

The multinational company where the study took place, in the area of metalworking, is characterised by workstations that ensure the mass production of parts of various sizes, which require repetitive movements and several hours in the standing position. As a result of the size of the parts produced, the space at the workstations is, in some situations, reduced, making it difficult to adopt correct postures.

The data collection instrument used was a self-completion questionnaire composed of two parts: sociodemographic and clinical characterization and the European Health Literacy Survey (HLS-EU), in the version translated and validated for the Portuguese population (HLS-EU-PT), with permission of the authors [3].

The HLS-EU is composed of 47 questions, designed according to a conceptual model that incorporates three important domains of health, namely Health Care (16 questions), Health Promotion (16 questions) and Disease Prevention (15 questions), and four levels of information processing, namely accessing, understanding, appraising and applying, which prove to be essential for decision making [3].

In the translation and validation for the Portuguese population, in order to test the correct calculation of the indices and ensure the comparison between them, the four calculated indices were standardised on a metric scale varying between 0 and 50, in which 0 represents the minimum possible health literacy level and 50 the maximum possible health literacy level [3].

Taking the above into consideration, the following cut-off points were identified for the four levels: scores equal to or lower than 25 points correspond to inadequate health literacy; scores between 25 and 33 points to problematic health literacy; scores between 33 and 42 points to sufficient health literacy; and scores between 42 and 50 correspond to excellent health literacy [3].

In the original 47-item scale, when validated for the Portuguese population, Cronbach’s alpha coefficients ranged between 0.90 and 0.96 [3]. In this study, Cronbach’s alpha coefficients ranged between 0.86 and 0.95.

The study was approved by the Ethics Committee under number 1247/2020 and authorised by the company’s management. At the beginning of the data collection, the questionnaires corresponding to the number of workers in the different sectors of the company were delivered and, subsequently, collected on site upon prior scheduling and availability of those responsible. In addition to the written information, which was attached to the questionnaire, at that moment, the research was also presented in person to the workers. Following the objectives’ clarification, the workers were free to decide on their participation and fill out the questionnaire, placing it in a closed envelope in case of participation. Data were collected between 14 April and 7 May 2021. All workers who agreed to participate in the study were asked to give their informed consent.

For data analysis, descriptive and inferential statistics were used with the help of the IBM Statistical Package for the Social Sciences (SPSS), version 26.0 (Armonk, New York, NY, USA). Quantitative variables were presented as median, mean and standard deviation (±), with a confidence interval of 95%.

Data normality was previously verified by the Shapiro–Wilk test. Considering the nature of the variables, to understand their distribution, the Mann–Whitney U test, Kruskal–Wallis test and Spearman’s correlation were used, considering the significance level of 5% (*p* < 0.05).

## 3. Results

### 3.1. Sociodemographic and Clinical Characterisation of the Participants

The study included 161 participants whose sociodemographic and clinical characteristics are explained in Table 1.

In relation to muscle and joint pain, the body sites most frequently mentioned by participants were the lumbar spine (*n* = 46), upper limbs (*n* = 32) and lower limbs (*n* = 21).

### 3.2. Health Literacy

Following the use of the HLS-EU-PT, the levels of health literacy are presented in Table 2.

### 3.3. Relationship of Health Literacy with Sociodemographic Variables

The associations between the sociodemographic variables and the health literacy indices are described in Table 3.

There is a weak association between age and the General and Disease Prevention Literacy indices (correlations of −0.168 and −0.157, respectively), i.e., the younger the age, the higher the value of the two indices. After noting that health literacy indices depend on the education level of the workers, it was necessary to better explore this variable. To compare the mean indices of the three levels of education (three pairs), we used the Mann–Whitney test (independent samples), so the significance level adjusted by the Bonferroni correction was 5%/3 ≈ 1.67%. It was then concluded that for all indices, the mean index of participants with basic education was lower than that of participants with secondary education.

### 3.4. Relationship of Health Literacy with Clinical Variables

The associations between clinical variables and health literacy indices are explained in Table 4.

With regard to body mass index (BMI) and the indices of General Literacy, Disease Prevention Literacy and Health Promotion Literacy, there is a weak and inverse correlation (−0.241, −0.201 and −0.252, respectively), that is, the higher the BMI, the lower the value of the indices.

In relation to disease diagnosis, a comparison of descriptive measures and the *p*-value of the Mann–Whitney test showed that all mean indices are higher for workers who have no diagnosed disease. Similarly, participants who did not take daily medication all showed higher literacy indices.

With regard to symptoms, the scores for General Literacy, Disease Prevention Literacy and Health Promotion Literacy were higher in workers who did not have joint pain, muscle pain, feelings of sadness, or memory changes that interfered with daily life.

**Figure 1:**
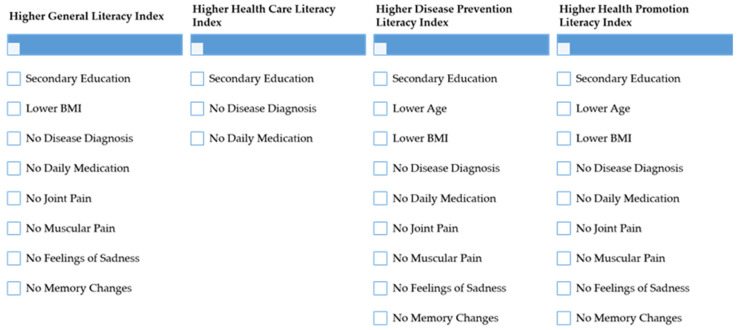
Variables associated with higher levels of health literacy. Source: Authors. BMI—body mass index.

Figure 1 summarises the variables that are associated with higher health literacy scores.

## 4. Discussion

From the analysis of the sociodemographic variables, we found that most of the workers who participated in the study were male, married and predominantly had secondary education, which corroborates the national data [18,19]. Regarding the body mass index, an average value of 26.1 kg/m^2^ was found, thus indicating that, on average, individuals were in a pre-obese state, which is in line with the data published for the Portuguese population [20]. More recently, in a survey conducted in 2020, 75.5% of the participants were overweight and 48.0% were pre-obese [21].

Regarding the clinical variables, 29.8% of the workers had at least one diagnosed disease, especially arterial hypertension and dyslipidaemia. Following the predominance of these two pathologies, 36.0% of the workers have instituted antihypertensives and antidyslipidaemia medications as daily therapy. The fact that we have a sample in which the state of pre-obesity predominated may be predictive of the existence of these two diagnosed diseases since hypertension and dyslipidaemia are comorbidities associated with obesity [20]. Authors of a study conducted in Portugal found that 47.9% of individuals had hypertension, followed by 11.2% of individuals with diabetes mellitus. In the same study, antihypertensives were the most commonly used medications (32.7%), followed by antidiabetics (10.2%) [21].

With regard to the symptoms, we highlight the visual impairment (36.6%), joint pain (31.7%), muscle pain (29.8%), feelings of sadness (28.0%), hearing impairment (26.7%) and memory changes that interfered with daily life (22.4%). In the case of muscle and joint pain, the lumbar spine, upper limbs and lower limbs stood out as anatomical regions, which is in line with what is described in the literature [22]. Following high work paces, associated with inappropriate postures and repetitive movements, the referred types of pain are often linked to work-related musculoskeletal disorders (WMSDs), which, according to the European Agency for Safety and Health at Work (EU-OSHA), affect mainly the back, neck, shoulders and upper limbs and may also affect the lower limbs [23].

It should be noted that in the company under study, prolonged standing, repetitive movements of the upper limbs, weight and the size/dimension of the parts produced affected the spine, upper and lower limbs in particular.

Regarding Health Literacy, in what concerns the average indexes, the Health Promotion Literacy index showed the lowest average (29.5), reinforcing the need for professionals to invest in this component.

In this context, the SNRN, based on the assumption that knowledge is constructed and integrated by each person, identifies the presence or fragility of this knowledge in its approach. Thus, in case of a knowledge deficit or fragility, the SNRN’s function is to empower, in the context of health education, involving knowledge construction, deliberation, and action, thus contributing to the person’s empowerment at the Health Literacy level. This empowerment provided by the SNRN intervention will be reflected in knowledge optimisation, increased autonomy and decision-making capacity, thus qualifying workers to actively manage their health conditions [12].

It is known that the prevention and treatment of various diseases, as well as WMSDs, requires the intervention of several health professionals, including the SNRNs. The latter, who often carry out their professional activity in companies similar to the one under study, are in a privileged position to promote Health Literacy by investing in health promotion and prevention of complications [9], which are still two weak areas.

In a study carried out in Portugal, about 61.0% of the population presented a problematic or inadequate level of General Health Literacy, with worrying values in the sub-dimension Health Promotion, in which 60.2% of the population presented an inadequate or problematic level of Health Literacy [7].

Other authors, in their study, found an inadequate level of literacy regarding the dimensions of Disease Prevention and Health Promotion [24].

In the analysis of the association between the sociodemographic and clinical variables in relation to education, namely between participants with basic education and participants with secondary education, it was confirmed that the mean index of workers with secondary education was higher than that of workers with basic education, for all literacy indexes. Younger workers had better General Literacy and Disease Prevention Literacy scores. Previous studies have confirmed that younger people and people with higher levels of education had higher levels of health literacy [3,7,25,26].

When investigating the relationship between clinical variables and Health Literacy, it was confirmed that all health literacy scores were, on average, higher among workers who had no diagnosed illness and took no medication. On the other hand, the lower the BMI, the higher the scores for General Literacy, Disease Prevention Literacy and Health Promotion Literacy.

With regard to symptoms, not experiencing muscle and joint pain, experiencing feelings of sadness and memory changes were significantly associated with higher rates of General Literacy, Disease Prevention Literacy and Health Promotion Literacy.

Following the results obtained in this study and in other similar studies, there is an urgent need to increase the levels of health literacy among the working-age population. Given the scope of the working-age population working in companies, this may become a privileged field of action for the SNRN in order to also simultaneously invest more deeply in the descriptive statements “Health Promotion” and “Prevention of Complications” [9].

In order to increase the levels of health literacy and, consequently, improve the symptomatology that workers often present, it is a priority to define programmes and initiatives to promote health literacy, contemplating not only the aspects that are directly linked to health but also those related to working conditions [7].

When this study was conducted, it was found that, when symptoms existed and were expressed by the workers, they often referred to muscle and joint pain, particularly in the spine and upper and lower limbs, in many situations related to WMSDs. In fact, musculoskeletal pain is one of the most commonly reported symptoms by workers from different contexts, and, according to some authors, physical exercise can largely contribute to preventing and reducing these symptoms [27].

Within companies, the investment in good physical activity and exercise practices, using innovative solutions, is a strategy capable of promoting healthier behaviours [23]. The risks of inactivity are commonly described in the literature; hence, the adoption of healthier lifestyles, where physical activity is included, is a key intervention [27].

In this sense, and in line with their specific competences [8], SNRN design and implement programmes adjusted to the needs and particularities of each worker’s professional exercise/workplace. In the scope of these programmes, the following stand out: Occupational Gymnastics Programmes; Physical Exercise Programmes; and Postural Education Programmes.

Occupational gymnastics programmes, also known as occupational physical activity, aim at promoting workers’ health by addressing the three components of the human being, namely the physical, psychological, and social components [27,28]. By preventing WMSDs and normalising body functions, physical activity at work also allows for moments of relaxation and socialisation, which are essential for interpersonal relationships.

With regard to physical exercise programmes, this type of exercise being considered therapeutic, it is characterised by a set of physical movements, postures or activities performed in a systematic and planned way, and which allow providing clients with the necessary means to mitigate or prevent impairments of body functions and structures, restore or improve activity and participation, prevent or reduce health-related risk factors and optimise overall health and sense of well-being [29,30,31].

Finally, postural education programmes should be adapted to the functions of the worker and the characteristics of the workstation, so it is important to previously perform a postural evaluation of the worker’s functions and of the workstation in order to obtain data that may be susceptible to change, with a view to reducing the worker’s complaints. This aspect is extremely relevant since the main factors associated with the development of musculoskeletal disorders are inadequate environmental and ergonomic factors, including inadequate body posture.

The presence of SNRN in the work contexts makes it possible for these programmes to be personalised and to take into consideration the specificities of the workplace and the complaints expressed by the worker.

Additionally, given the results obtained in this research, namely the predominance of workers with a BMI characterising a pre-obesity state and the presence of diseases that are associated with eating habits, specifically hypertension and dyslipidaemia, it also becomes important to invest in the promotion of self-care as a key strategy for the adoption of healthy lifestyle habits and the management of the existing disease itself. Promoting health literacy and empowering self-care not only adds benefit to the individual worker’s health but is also considered an essential factor in preventing work absenteeism and early turnover, improving job performance and ensuring human resources in work settings [32].

Health literacy improves self-management of health and promotes informed self-care behaviours and healthy lifestyles and active participation in the care of one’s own health, thus preventing diseases [32].

In this context, increasing health literacy at a young age has the potential to reduce health problems in the future, maintain long-term health and prevent workers from leaving the company for health reasons [33,34].

Following on from the above, in addition to face-to-face sessions on physical exercise, the provision of informative material on postures to be adopted, considering the workstation in which they operate, and on good health habits, are also relevant.

Still, within the scope of the symptoms, the participants in this study mentioned as frequent the decrease in vision and hearing, which, although they are irreversible changes, require the intervention of the SNRN in order to promote the adaptation of workers to the deficits and ensure that the workstations are adjusted to their limitations.

In the case of WMSDs already having irreversible clinical repercussions, the SNRN also has an important role in teaching, instructing and training adaptive techniques, maximising the worker’s performance and well-being.

### Limitations

This study has some limitations. First, the small sample size, the fact that the research was only conducted in one work context and the use of a single data collection strategy prevent the generalisation of results. Second, the type of study carried out (cross-sectional) makes it difficult to determine the cause–effect relationships between the variables. Third, the fact that the research involved participants’ self-reports and that data collection took place during one of the phases of the pandemic by COVID-19 may have influenced some of our findings; hence, the risk of response bias should be considered.

Thus, studies of other typologies, such as longitudinal designs, with a larger number of participants and carried out in different contexts, are suggested in order to contribute to the validation and generalisation of the results.

## 5. Conclusions

The results are in line with previous studies that suggest the need to invest in programmes and initiatives to promote health literacy, covering not only aspects related to health promotion but also those related to the prevention of complications associated with working conditions.

Although most of the workers in this study presented a good health status, they faced risks associated with their living and working conditions. The SNRN plays a key role in the adherence to healthy behaviours and in promoting active participation in self-management of health, thus empowering workers for self-care and healthy lifestyles while making them aware of the benefits and repercussions throughout the life cycle.

This becomes even more relevant as, in this study, low levels of health literacy were identified among workers with lower levels of education and older workers, as well as with the presence of risk factors for the development of cardiovascular diseases and with a higher incidence of chronic diseases.

In this context, and since health literacy is a fundamental resource for the prevention and reduction of risks for several non-communicable diseases, as well as for the optimisation of access to health and the involvement of workers, the definition of programmes and the adoption of strategies to enhance it are emergent.

## Figures and Tables

**Table 1:** Sociodemographic and clinical characterisation of the participants.

Gender *n* (%)	
Female	11 (6.8)
Male	150 (93.2)
Age (years) *n* (%)	
<20 years old	3 (1.9)
20–30 years old	32 (19.9)
30–40 years old	34 (21.1)
40–50 years old	33 (20.5)
50–60 years old	38 (23.6)
>60 years old	21 (13.0)
Marital status *n* (%)	
Single	55 (34.2)
Married/non-marital partnership	94 (58.4)
Divorced	9 (5.6)
Widower	3 (1.8)
Education *n* (%)	
Basic education	61 (37.9)
Secondary education	67 (41.6)
Higher Education (university/polytechnique/other)	33 (20.5)
Body mass index mean; median; std. dev.	26.1; 25.6; 3.6
Current disease diagnosis *n* (%)	
Yes	48 (29.8)
No	113 (70.2)
Diseases *n* (%)	
Hypertension	26 (54.2)
Dyslipidaemia	12 (25.0)
Diabetes mellitus	4 (8.3)
Depression	4 (8.3)
Coronary heart disease	2 (4.2)
Daily medication *n* (%)	
Yes	58 (36.0)
No	103 (64.0)
Type of medication *n* (%)	
Antihypertensives	28 (17.4)
Antidyslipidemics	17 (10.6)
Antidepressant	9 (5.6)
Antacid	9 (5.6)
Anxiolytic	7 (4.3)
Antidiabetic	4 (2.5)
Symptoms *n* (%)	
Vision impairment	59 (36.6)
Joint pain	51 (31.7)
Muscular pain	48 (29.8)
Feelings of sadness	45 (28.0)
Hearing impairment	43 (26.7)
Memory changes	36 (22.4)
Headaches	24 (14.9)
Dizziness	14 (8.7)
Loss of urine	9 (5.6)
Imbalances	5 (3.1)
Difficulty in walking	4 (2.5)

Source: Authors. Std. dev.—standard deviation.

**Table 2:** Participants’ levels of health literacy.

	Literacy Indices *	General Literacy Index (GI)	Health Care Literacy Index (HCI)	Disease Prevention Literacy Index (DPI)	Health Promotion Literacy Index (HPI)
Coefficients					
Minimum		13.5	19.8	6.0	2.1
Maximum		50.0	50.0	50.0	50.0
Average		30.3	31.4	30.1	29.5
Standard deviation		6.2	6.0	7.2	7.4

Source: Authors. * Scores of 25 or less = inadequate health literacy; scores between 25 and 33 points = problematic health literacy; scores between 33 and 42 points = sufficient health literacy; scores between 42 and 50 = excellent health literacy.

**Table 3:** Association between sociodemographic variables and health literacy indices.

	Literacy Indices	General Literacy Index (GI) *p*-Value	Health Care Literacy Index (HCI) *p*-Value	Disease Prevention Literacy Index (DPI) *p*-Value	Health Promotion Literacy Index (HPI) *p*-Value
Variables					
Gender		0.501 *	0.770 *	0.687 *	0.333 *
Age		0.033 ^†^	0.126 ^†^	0.046 ^†^	0.052 ^†^
Education		0.002 ^‡^	0.023 ^‡^	0.008 ^‡^	0.002 ^‡^
Marital status		0.157 ^‡^	0.177 ^‡^	0.260 ^‡^	0.140 ^‡^

Source: Authors. * Mann–Whitney test. ^†^ Spearman’s correlation. ^‡^ Kruskal–Wallis test.

**Table 4:** Association between clinical variables and health literacy indices.

	Literacy Indices	General Literacy Index (GI) *p*-Value	Health Care Literacy Index (HCI) *p*-Value	Disease Prevention Literacy Index (DPI) *p*-Value	Health Promotion Literacy Index (HPI) *p*-Value
Variables					
Body mass index		0.002 *	0.058 *	0.011 *	0.001 *
Disease diagnosis		0.002 ^†^	0.023 ^†^	0.001 ^†^	0.008 ^†^
Daily medication		<0.001 ^†^	0.008 ^†^	<0.001 ^†^	0.001 ^†^
SYMPTOMS					
Vision impairment		0.217 ^†^	0.273 ^†^	0.403 ^†^	0.139 ^†^
Joint pain		0.027 ^†^	0.191 ^†^	0.029 ^†^	0.032 ^†^
Muscular pain		0.024 ^†^	0.051 ^†^	0.018 ^†^	0.025 ^†^
Feelings of sadness		0.008 ^†^	0.061 ^†^	0.043 ^†^	0.002 ^†^
Hearing impairment		0.209 ^†^	0.602 ^†^	0.396 ^†^	0.158 ^†^
Memory changes		0.012 ^†^	0.322 ^†^	0.012 ^†^	0.005 ^†^
Other types of pain		0.347 ^†^	0.844 ^†^	0.406 ^†^	0.096 ^†^
Dizziness		0.304 ^†^	>0.999 ^†^	0.143 ^†^	0.240 ^†^
Loss of urine		0.291 ^†^	0.253 ^†^	0.258 ^†^	0.243 ^†^
Imbalances		0.375 ^†^	0.461 ^†^	0.334 ^†^	0.553 ^†^
Difficulty in walking		0.696 ^†^	0.753 ^†^	0.393 ^†^	0.527 ^†^

Source: Authors. * Spearman’s correlation. ^†^ Mann–Whitney test.

## Data Availability

The data that support the findings of this study are available from the corresponding author upon reasonable request.
